# Supplementary material for: Phosphoproteins Involved in the Inhibition of Apoptosis and in Cell Survival in the Leiomyoma
Source: J Clin Med. 2019 May 16;8(5):691. doi: 10.3390/jcm8050691 (PMC6572112; doi:10.3390/jcm8050691)
Supplement: Supplementary file 1 [file jcm-08-00691-s001.pdf]

Supplementary data

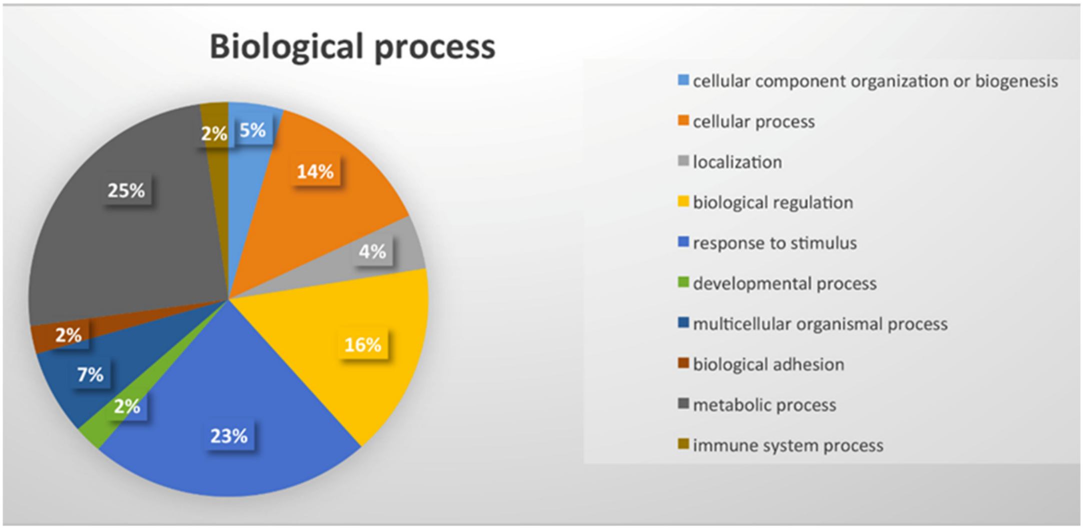

Figure S1. PANTHER classification of identified proteins in the leiomyoma according to their biological process.

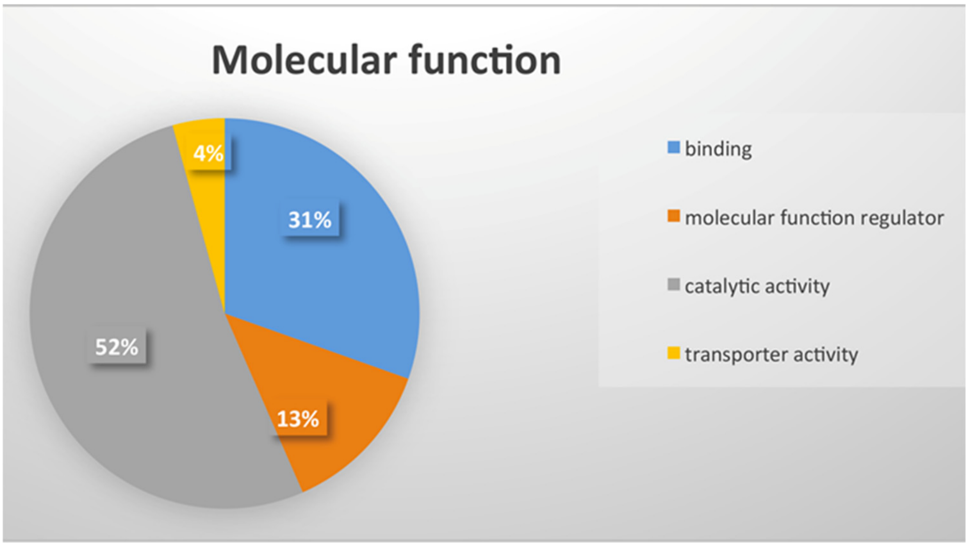

Figure S2. PANTHER classification of identified proteins in the leiomyoma according to their molecular function

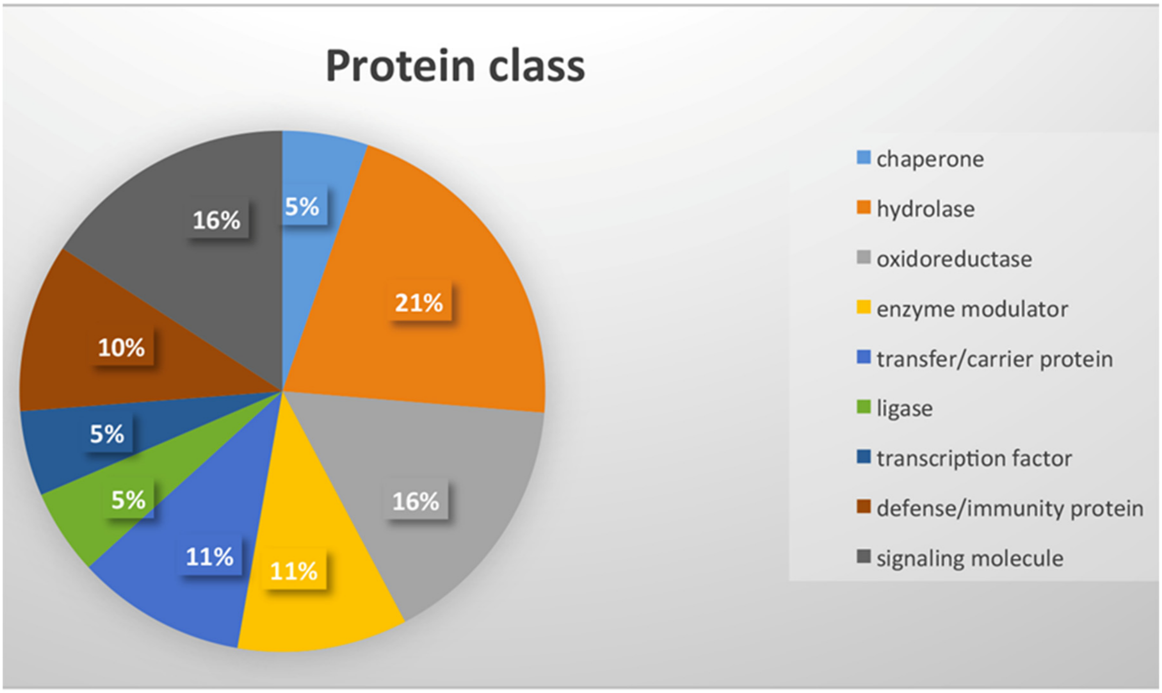

Figure S3. PANTHER classification of identified proteins in the leiomyoma according to their protein class.

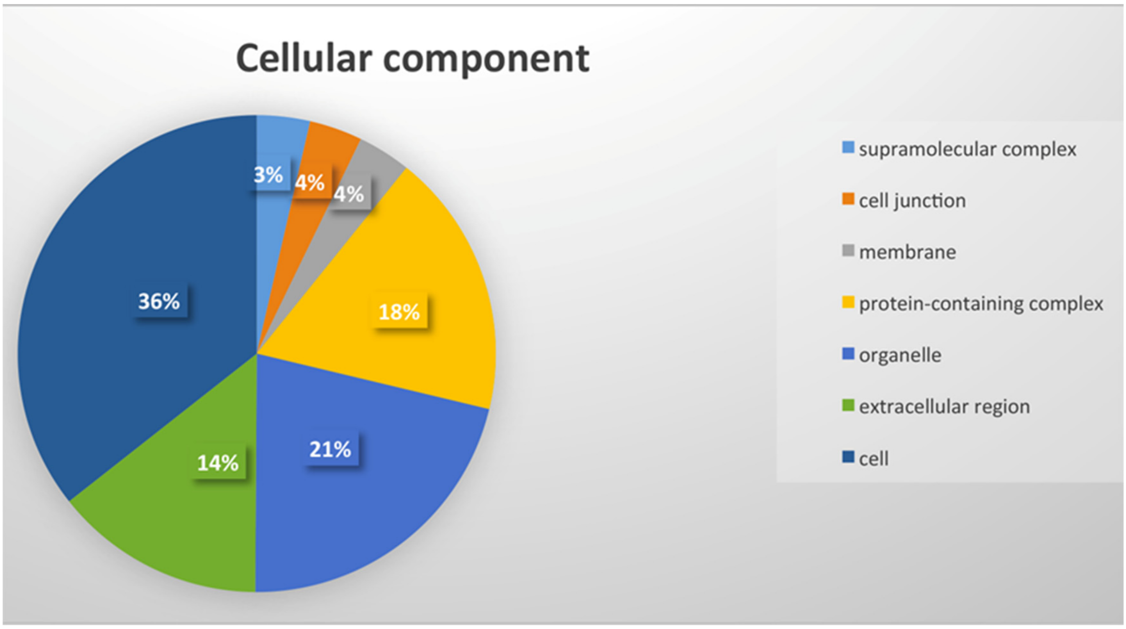

Figure S4. PANTHER classification of identified proteins in the leiomyoma according to their cellular component.

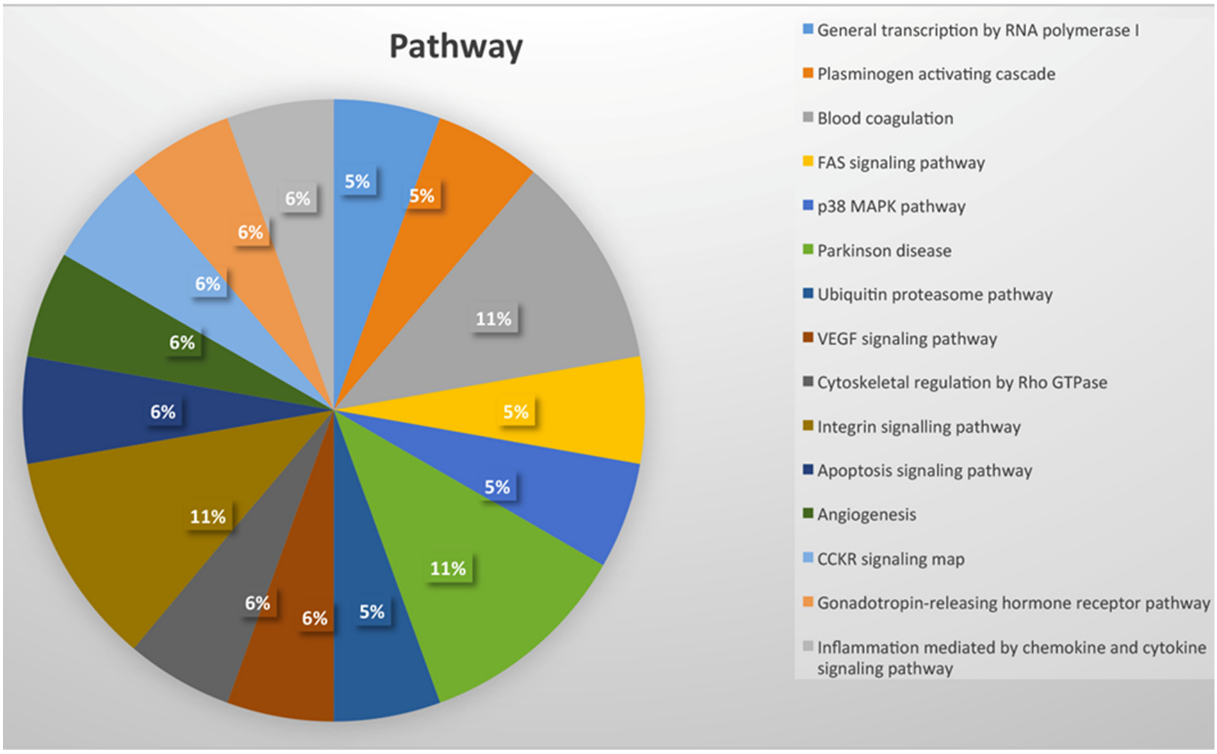

Figure S5. PANTHER classification of identified proteins in signaling pathways.

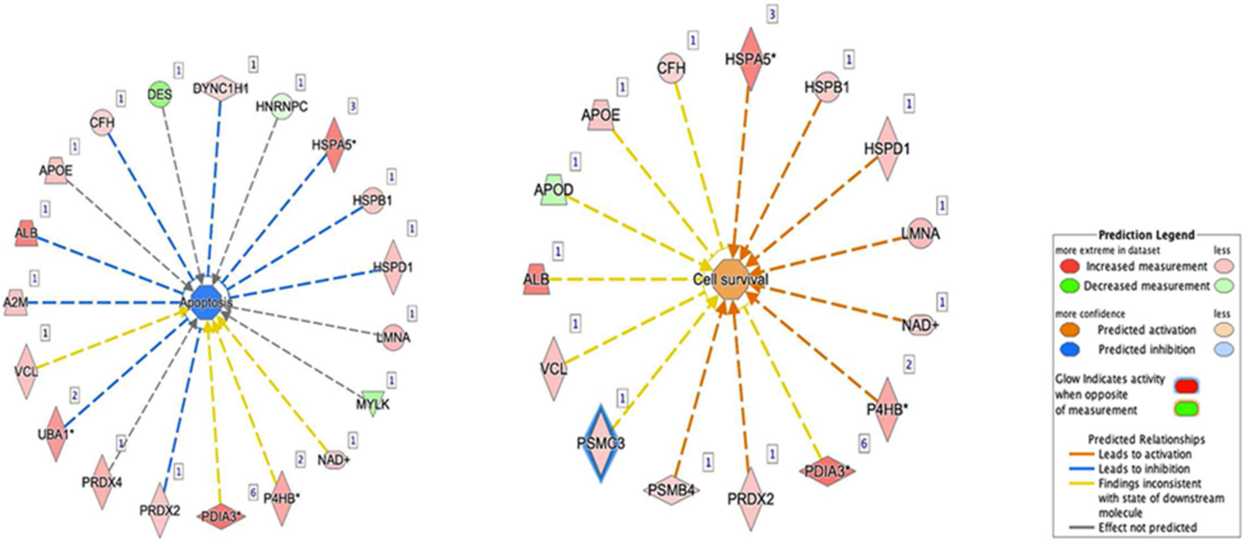

Figure S6. Network build up from one of the most significant bio-functions: 1. Apoptosis, 2. Cell survivor.
